# Effectiveness of school-based physical activity and nutrition interventions with direct parental involvement on children’s BMI and energy balance-related behaviors – A systematic review

**DOI:** 10.1371/journal.pone.0204560

**Published:** 2018-09-27

**Authors:** Sacha R. B. Verjans-Janssen, Ilona van de Kolk, Dave H. H. Van Kann, Stef P. J. Kremers, Sanne M. P. L. Gerards

**Affiliations:** 1 Department of Health Promotion, School of Nutrition and Translational Research in Metabolism (NUTRIM), Maastricht University, Maastricht, The Netherlands; 2 School of Sport Studies, Fontys University of Applied Sciences, Eindhoven, The Netherlands; TNO, NETHERLANDS

## Abstract

**Background:**

The aims of this systematic review were to study the effectiveness of primary school-based physical activity, sedentary behavior and nutrition interventions with direct parental involvement on children’s BMI or BMI z-score, physical activity, sedentary behavior and nutrition behavior and categorize intervention components into targeted socio-cognitive determinants and environmental types using the Environmental Research framework for weight Gain prevention.

**Methods:**

In March 2018, a systematic search was conducted in four electronic literature databases. Articles written in English about effectiveness studies on school-based interventions with direct parental involvement targeting 4–12 year olds were included. Interventions with indirect parental involvement, interventions not targeting the school environment, and pilot studies were excluded. Study and intervention characteristics were extracted. Study quality and study effectiveness were assessed and effect sizes (Cohen’s d) were calculated for the outcome measures. Types of socio-cognitive factors and environmental types targeted were distinguished.

**Results:**

In total, 25 studies were included. Most studies on BMI or BMI z-score, physical activity and sedentary behavior found favorable results: 61.1%, 81.1% and 75%, respectively. Results regarding nutrition behavior were inconclusive. Methodological study quality varied. All interventions targeted multiple environmental types in the school and family environment. Five targeted socio-cognitive determinants (knowledge, awareness, attitude, self-efficacy and intrinsic motivation) of the children were identified. No consistent pattern was found between either type of environment targeted, number of type of environment targeted, or the child’s targeted socio-cognitive determinants and intervention effectiveness.

**Discussion:**

School-based interventions with direct parental involvement have the potential to improve children’s weight status, physical activity and sedentary behavior. Based on the results, it is recommended that school-based interventions with direct parental involvement target more than one EBRB, last at least one year, and focus particularly on the physical and social environment within both the school and the family environment.

## Background

Since 1980, overweight and obesity prevalence rates among children have increased rapidly [1]. Although many local, national and international efforts have been implemented to reduce overweight and obesity [2, 3], their prevalence among children and adolescents is still alarmingly high and increasing. In 2013, 23.8 percent of boys and 22.6 percent of girls in developed countries were overweight or obese [1]. Unhealthy eating behaviors, low levels of physical activity and a sedentary lifestyle are important causes of overweight and obesity [4]. These individual energy balance-related behaviors (EBRBs) are influenced by multiple factors, such as the environment to which a child is exposed [5, 6].

Two important environmental settings affecting children’s EBRBs are the family environment and the school environment. Since schools have a large reach [7], many school-based interventions have been developed with the aim to promote healthy EBRBs of primary school children [3]. Considering the important influence of parents on children’s EBRBs, the WHO School Health Promotion Framework advocates parental involvement in these school-based interventions [8]. As a result, a larger number of school-based interventions with parental involvement are being implemented [9].

Although the importance of parental involvement in school-based interventions is recognized [8, 10, 11], the evidence regarding the effectiveness of school-based, overweight-prevention interventions in which the parents were involved is inconclusive. A systematic review conducted in 2010 on combined community or school and home-based, obesity-prevention interventions found the results of 7 of the 15 included studies to be effective regarding nutrition behavior, physical activity behavior, sedentary behavior, weight status, or health risk factors [12]. A more recent review on school-based, overweight-prevention interventions in which the family environment was also targeted showed that 8 studies were effective regarding weight-related outcomes, whereas 19 studies had mixed results and 14 studies had ineffective results [13]. Also, the evidence regarding the additional effectiveness of parental involvement in school-based interventions remains uncertain [14, 15]. The explanations for this were an inadequate number of studies on school-based interventions with parental involvement [14, 15] and the mixed results reported by these studies [15].

These previous systematic reviews included both school-based interventions with *direct* or *indirect* parental involvement. Their results did show that school-based physical activity and nutrition interventions with *direct* parental involvement (e.g. parents were educated on health-related topics via group sessions) were more likely to be effective than school-based interventions in which parents were *indirectly* involved (e.g. parents were sent a newsletter). However, more research is needed to confirm these results [14].Along with different settings (e.g. school and home), socio-ecological frameworks suggest the importance of targeting different types within these environments [16–18]. According to the Environmental Research framework for weight Gain prevention (EnRG) [17] the physical, social, economic and political environment influences children’s EBRBs at the micro- and macro-levels [16], either directly or mediated by socio-cognitive factors. The framework can be used to disentangle the determinants targeted in interventions (individual socio-cognitive determinants and the different environmental types within different settings) that may have been important for changing children’s EBRBs. To our knowledge, no systematic review on the effectiveness of school-based physical activity and nutrition interventions with direct parental involvement has identified targeted child’s socio-cognitive determinants and environmental types within the school and family environment in order to explore a pattern between these factors and intervention effectiveness. Including this contextual information can contribute substantially to the understanding of intervention effectiveness. The aims of this systematic review were to study the effectiveness of school-based physical activity and nutrition interventions with direct parental involvement regarding children’s weight status and EBRBs and to categorize the intervention components into distinct types of socio-cognitive factors and different environmental types targeted using the EnRG framework [17].

## Methods

Although the protocol of this systematic review was not registered before conduct of the study, procedures were protocoled and described in detail here to enhance transparency and reproducibility. A literature search was performed in order to conduct two systematic reviews: one systematic review regarding *preschool* interventions with a direct parental involvement component targeting the EBRBs of children aged 2–4 years (manuscript in preparation), and one systematic review regarding *primary school* interventions with direct parental involvement targeting the EBRBs of children aged 4–12 years. The latter review is the current study. The literature search was conducted by two reviewers (SV and IvdK) in *Pubmed*, *Web of Science*, *PsychInfo* and *ERIC* in June 2016 and updated in June 2017 and March 2018. A list of relevant categories and related search terms and keywords was prepared (Table 1), consisting of six categories: (1) intervention participants, (2) intervention target behaviors, (3) school environment, (4) family environment, (5) intervention, and (6) effectiveness studies. As an illustration, the search strategy used in Pubmed is presented in S1 Table.

**Table 1:** Categories and terms of the search strategy.

**Category 1: Intervention participants**
Child(ren), preschool child(ren), minor(s), toddler(s), infant(s)
**Category 2: Intervention target behaviors**
Motor activity, physical activity, physical activities, sedentary behavior, lifestyle, energy balance, diet(s), dietary, food, nutrition, (un)healthy food, (un)healthy eating, energy intake
**Category 3: School**
Nursery, nurseries, child day care center(s), day care(s), preschool(s), kindergarten(s), playgroup(s), school(s), primary school(s), school-based, school-centered
**Category 4: Family**
Parent(s), father(s), mother(s), caregiver(s), family, families, family based, home (based), parental
**Category 5: Intervention**
Intervention(s)
**Category 6: Effectiveness studies**
Evaluation(s) (study), effect(s), effective(ness), effectivity, pre-post-test(s)

### Inclusion criteria

Studies were included if they:

1. investigated the effectiveness of a school-based intervention targeting physical activity behavior (PA), sedentary behavior (SB) and/or nutrition behavior (NB);
2. considered the effects on children’s Body Mass Index (BMI), the BMI z-score (BMI adjusted for age and gender) and/or PA, SB and NB;
3. targeted children aged 4 to 12 years attending primary school;
4. studied a school-based intervention which consisted of at least one of the following types of interventions: (a) changes to the school’s physical environment, e.g. providing fruit or vegetables at school, or creating an activity-friendly playground; (b) changes to the school’s social environment, e.g. training school staff about health promotion, or the implementation of activity breaks by teachers; (c) changes to the school’s policies, e.g. rules about fruit and vegetable consumption at school or active transportation to school; or (d) economic support for the school, e.g. a budget for implementing activities promoting physical activity or providing fruit and vegetables;
5. studied a school-based intervention that *directly* involved parents. The definition of ‘direct parental involvement’ by Hingle et al. [14] was used: requesting parents to attend energy balance-related education sessions, e.g. workshops or lessons promoting physical activity, improving children’s diet or reducing sedentary behavior; or asking parents to attend or participate in family behavior counseling or parent training sessions (14). These sessions could have been conducted at home (in group sessions or one-on-one) or at a different location (at school, for example);
6. were written in English.

### Exclusion criteria

A study was excluded if:

1. the intervention only *indirectly* involved parents, as defined by Hingle et al. [14]: (1) sending newsletters or information sheets to parents; (2) inviting parents to attend a health-related information evening; or (3) giving children homework that should be made with the help of their parents;
2. the intervention did not target the school environment (i.e. change the normal school’s routine);
3. the intervention was exclusively aimed at a particular subpopulation, e.g. overweight primary school children;
4. it was defined as a pilot study by the study authors. This was done because the aim of pilot studies is to test an intervention’s feasibility instead of its effectiveness.

### Study selection

After removing the duplicates, the retrieved articles were screened independently on title/abstract by the two researchers (SV and IvdK). The remaining articles were screened as full text to assess the eligibility of the studies, based on the inclusion and exclusion criteria, determined *a priori*. Again this was done, independently, by two researchers (SV and IvdK). An overall agreement between the researchers of 74.6% existed. Discrepancies were discussed until consensus was reached. If no consensus was reached, a third researcher (SG) independently assessed the eligibility of the studies. The third researcher was consulted for four articles.

### Data extraction

The PRISMA statement was used in writing this systematic review [19] (S1 File). One researcher (SV) conducted the data extraction regarding the study characteristics, intervention characteristics, and study effectiveness using predefined forms. The information on the following study characteristics was extracted: study design, setting, number of schools participating in the study, the timeframe in which follow-up measurements were made, number of participants, drop-out rates, characteristics of the participants and outcome measures.

The methodological quality of the studies was assessed using the quality assessment instrument of the Effective Public Health Practice Project (EPHPP) [20]. This instrument can be used to assess the quality of quantitative studies with a variety of study designs [20]. The studies were rated on six key quality components: selection bias, study design, confounders, blinding, data collection methods, withdrawals and drop-outs. Each of these quality components was rated ‘weak’, ‘moderate’ or ‘strong’. For example, a strong score was given when the study design was described as an RCT or a controlled clinical trial. An overall score was given based on the scores of the six quality components. The overall quality was rated: ‘strong’ when there were no weak and at least four strong ratings for the six quality components; ‘moderate’ when only one quality component was rated as weak; and ‘weak’ in case there were two or more weak ratings [20]. Two researchers (SV and IvdK) independently rated the quality of the articles. The interrater reliability was 72.1%. Differences were the result of different interpretations of the studies. The researchers compared their quality scoring results and reached consensus by discussion.

The effects on BMI, BMI z-score, physical activity behavior, sedentary behavior, and nutrition behavior were described. Study effectiveness was regarded as positive when all results regarding a particular outcome (e.g. BMI and BMI z-score) showed a statistically significant improvement for the intervention group. Study results were considered mixed when at least one finding of a particular outcome was statistically significant in favor of the intervention group but the other findings were not (e.g. a statistically significant improvement in fruit intake and ineffective or negative results regarding vegetable intake). The results were considered negative, when the results were statistically significant in favor of the control group. An intervention was considered ineffective when there were no statistically significant results for either the intervention or the control group.

Where possible, Cohen’s d effect sizes were calculated to indicate the standardized difference between the means of the intervention and control groups for the different outcome measures [21]. In case of multiple intervention arms, only the effects of the intervention arm that was school-based and included parental involvement were recorded. For studies without a control group, Cohen’s d was calculated by dividing the mean change in the outcome measure by the standard deviation of the baseline value. Lipsey’s cut-off points [22] were used to classify the magnitude of the effect: an effect size below 0.32 was considered ‘small’, between 0.33 and 0.55 ‘moderate’ and 0.56 or more ‘large’ [22]. When information required for calculating an effect size was missing (as was the case in five studies), a request was sent to the authors to provide the missing information. One author responded to this request.

Regarding the interventions, information was extracted on the intervention duration and the behavior targeted by the intervention. In addition, the different types of socio-cognitive determinants of the children (knowledge, awareness, attitude, subjective norms, self-efficacy, intrinsic motivation) and the different environmental types (political, economic, physical and sociocultural) affecting the child were distinguished, according to the EnRG framework by Kremers et al. [17]. Examples of the environmental types are the parental rules regarding the child’s dietary behavior (political), the costs of healthy foods in the school canteen (economic), the availability of play equipment during school breaks (physical) and the stimulation of physical activity behavior at school by the teachers (sociocultural).

## Results

**Fig 1:**
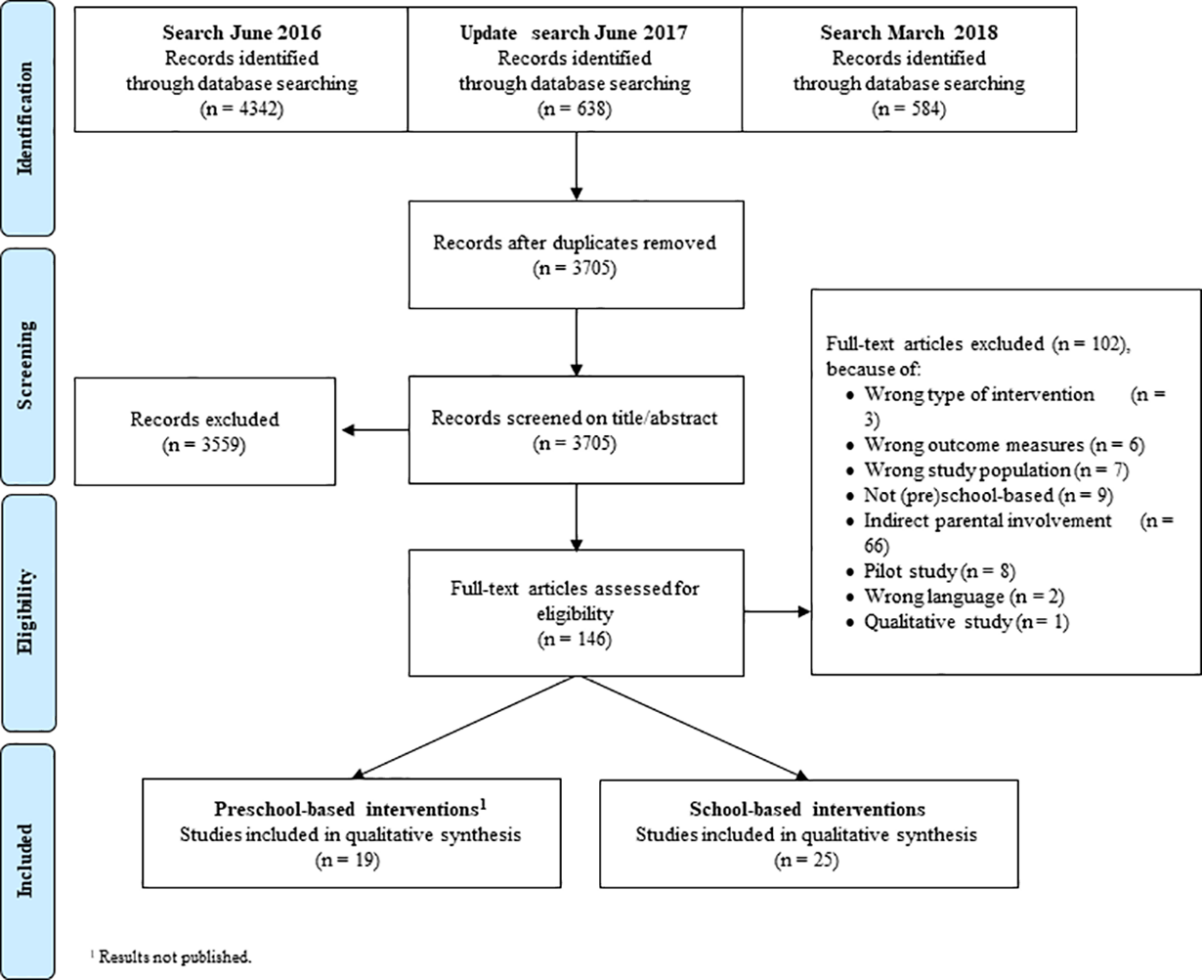
Flowchart of study selection.

The literature search resulted in a total of 5,564 studies and after removal of duplicates, a total of 3,705 studies remained. After screening on title and abstract, 146 records were assessed for eligibility by reading the full text. Finally, 25 studies describing the effectiveness of primary school-based interventions with direct parental involvement were included (Fig 1). The main reason for exclusion was that parents were only indirectly involved (n = 66): in most cases parents only received newsletters or information documents (n = 31).

### General study characteristics

The 25 included studies described the effectiveness of 24 school-based interventions with direct parental involvement. Of the 25 included studies, ten were randomized controlled trials [23–32], eleven were quasi-experimental studies [33–43], three had a pretest-posttest design [44–46] and one study had a repeated cross-sectional design [32] (Table 2). Most interventions (n = 9) were conducted in the USA [26, 33, 35–37, 40, 42, 43, 45]. The interventions were conducted between 1992 and 2015. The number of schools participating in the studies ranged from 1 [37] to 38 [47]. All studies performed the follow-up measurements immediately at the end of the intervention. One study conducted longer-term follow-up measurements (one year follow-up) [24]. The number of children participating in the study at baseline ranged from 97 [37] to 9867 [32]. Drop-out rates varied from 2% [25] to 48% [45]. Information on drop-out rates was missing in six studies [23, 35–37, 40, 43]. The average age of children participating in the included studies ranged from 5 [46] to 12 years [24]. Eighteen studies measured intervention effects on BMI or BMI z-score [23, 25, 26, 28–34, 36, 38, 39, 41, 43–45, 47]. Eleven studies measured PA [23, 26, 27, 29–32, 34, 39, 44, 46] and nine studies measured SB [26, 27, 30–32, 34, 35, 44, 46]. Twelve studies measured NB [23, 24, 26, 29, 31, 32, 36, 37, 40, 42, 44, 46].

**Table 2:** General characteristics of the included studies.

Authors	Study characteristics	Intervention characteristics	Population characteristics	Outcome measures			
	Study design,	Country, year,	No. of schools,	BMI (z-score)	Physical activity behavior	Sedentary behavior	Nutrition behavior
	follow-up,	duration,					
	sample size (dropout)	behavior targeted	mean age				
Alexander et al. (2014) [33]	Quasi-exp.	USA, 2011	2 Intervention schools	BMI (kg/m^2^) per BMI percentile subgroup^a^			
	6 months	6 months	2 Control school				
	N = 749 (25%)	PA, NB	*NR* (range: 6–8 yrs)				
Angelopoulos et al. (2009) [23]	RCT	Greece, 2005–2006	13 Intervention schools	BMI (kg/m^2^)^a^	MVPA (min/day)^b^		Fruit intake (exchanges/day)^b^
	14 months	12 months	13 Control schools	BMI z-score^a^			Vegetable intake (exchanges/day)^b^
	N = 646 (*NR*)	PA, NB	10.3 ± 0.4 yrs				Dairy intake (exchanges/day)^b^
							Fats and oils intake (exchanges/day)^b^
							Meat intake (exchanges/day)^b^
							Grains intake (exchanges/day)^b^
							Sweets and beverages intake (exchanges/day)^b^
Bacardí-Gascon et al. (2012) [44]	Pretest-posttest	Mexico, 2008–2010	4 Intervention schools	BMI (kg/m^2^)^a^	Outdoor play (h/day)^b^	Sitting (h/day)^b^	Fruit intake (portions/day)^b^
	24 months	6 months	*No control schools*	BMI z-score^a^	Physical education (h/week)^b^	TV watching (h/day)^b^	Vegetable intake (portions/ day)^b^
	N = 532 (10%)	PA, NB	8.5 ± 0.7 yrs		Supervised sports or dancing (h/week)^b^	Computer and video games (h/day)^b^	Sugar-sweetened beverages intake (portions/day)^b^
							Soda intake (portions/day)^b^
							Chocolate and candy intake (portions/day)^b^
							Snack intake (portions/day)^b^
Bere et al. (2006) [24]	RCT	Norway, 2001–2003	9 Intervention schools				Fruit and vegetable intake at school (portions/day)^b^
	8 months and 1 year and 8 months	6 months	10 Control schools				Fruit and vegetable intake (portions/day)^b^
	450 (18%)	NB	11.3 ± *NR* yrs				
Cao et al. (2015) [25]	cluster RCT	China, 2011–2013	8 Intervention schools	BMI z-score^a^			
	1, 2 and 3 years	34 months	8 Control schools				
	1854 (2%)	PA, NB	6.9 ± 0.3 yrs				
Centis et al. (2012) [34]	Quasi-exp.	Italy, 2008–2009	7 Schools	BMI (kg/m^2^)^a^	Time spent in outdoor activities (h/week)^c^	Time spent watching TV (h/week)^c^	
	8 months	5 months	(1 intervention-arm1 & control-arm)	BMI z-score^a^	Time spent in extra-curricular sports courses (h/week)^c^		
	209 (10%)	PA, NB	9.4 ± 0.3 yrs				
Chomitz et al. (2010) [45]	Pretest-posttest	USA, 2005–2007	12 Intervention schools	BMI z-score^a^			
	3 years	3 years	*No control schools*				
	3561 (48%)	PA, NB	7.7 ± 1.8 yrs				
Cong et al. (2012) [35]*	Quasi-exp.	USA, 2007–2008	2 Intervention schools			Sedentary behavior (hours of screen viewing/day)^c^	
	22 months	22 months	2 Control schools				
	N = 416 (*NR*)	PA, NB	6.7 ± 1.0 yrs				
Crespo et al. (2012) [26]	RCT	USA, 2003–2006	3 Intervention schools	BMI z-score^a^	PA behavior (PA behavior compared to other children of same age and sex with 1 = much less than others; 5 = much more than others)^c^	TV viewing (frequency of TV viewing while getting ready for school with 1 = never; 5 = always)^c^	Fruit and vegetable intake (servings/day)^c^
	1, 2 and 3 years	3 years	4 Control schools	BMI percentile for age and gender^a^	Team sports (no. of team sports participated in in past year)^c^		Snack intake (servings/day)^c^
	N = 392 (45%)	PA, NB	5.9 ± 0.9 yrs				Sugar-sweetened beverages intake (servings/day)^c^
							Water consumption (servings/day)^c^
Engelen et al. (2013) [27]	RCT	Australia, 2009–2010	6 Intervention schools		Light PA (min/day)^a^	Time spent sedentary (min/day)^a^	
	13 weeks	13 weeks	6 Control schools		MVPA (min/day)^a^		
	N = 221 (7%)	PA	6.0 ± 0.6 yrs				
Feng et al. (2016) [36]*	Quasi-exp.	USA, 2007–2008	2 Intervention schools	BMI percentile for age and gender^a^			Sugar-sweetened beverages consumption (oz/day)^c^
	4, 10, 16 and 22 months	22 months	2 Control schools				
	N = 555 (*NR*)	PA, NB	6.7 ± 1.0 yrs				
Hopper et al. (1996) [37]	Quasi-exp.	USA, *NR*	1 school				Cholesterol intake (mg/weekday)^c^
	12 weeks	10 weeks	(1 intervention-arm & 1 control-arm)				Saturated fat intake (mg/weekday)^c^
	N = 97 (*NR*)	PA, NB	8.9 ± 1.2 yrs				Fruit and vegetable intake (servings/weekday)^c^
							Grain and cereal intake (servings/ weekday)^c^
Jiang et al. (2007) [28]	RCT	China, *NR*	2 Intervention schools	BMI (kg/m^2^)^a^			
	3 years	3 years	3 Control schools				
	N = 2489 (3%)	PA, NB	8.3 ± 1.5 yrs				
Kain et al. (2004) [38]	Quasi-exp.	Chile, 2002	3 Intervention schools	BMI (kg/m^2^)^a^			
	8 months	6 months	2 Control schools	BMI z-score^a^			
	N = 3577 (14%)	PA, NB	10.6 ± 2.5 yrs				
Li et al. (2014) [39]	Quasi-exp.	China, 2012–2013	2 Intervention schools	BMI (kg/m^2^)^a^	Duration of MVPA (min/day)^b^		
	12 weeks	12 weeks	2 Control schools				
	N = 921 (7%)	PA, NB	10.4 ± 2.2 yrs				
Manios et al. (1999) [29]	RCT	Greece, 1992–1995	12 Intervention schools	BMI (kg/m^2^)^a^	Leisure-time MVPA (h/week)^c^		Energy intake (kcal/days)^c^
	3 years	3 years	9 Control schools				Total fat (g/day)^c^
	N = 579 (19%)	PA, NB	*NR*				Cholesterol (g/day)^c^
							Protein (g/day)^c^
							Carbohydrate (g/day)^c^
							Fiber (g/day)^c^
Müller et al. (2001) [46]	Pretest-posttest	Germany, 1996–1999	3 Intervention schools		PA (% Children performing daily PA)^b^	TV watching (h/day)^b^	Fruit and vegetables intake (% children with daily intake)^b^
	1 year	4 years	3 Control schools (waiting list control group)				
	N = 2440 (33%)	PA, NB	*NR* (range: 5–7 yrs)				
Prelip et al. (2012) [40]	Quasi-exp.	USA, 2009–2010	4 Intervention schools				Fruit intake (number of times/day)^b^
	10 months	10 months	2 Control schools				Vegetables intake (number of times/day)^b^
	N = 399 (*NR*)	NB	NR (range: 8–11 yrs)				
Sanigorski et al. (2008) [41]	Quasi-exp.	Australia, 2003–2006	10 Intervention schools (4 preschools and 6 primary schools)	BMI (kg/m^2^)^a^			
	3–4 years	3 years	16 Control schools (4 preschools and 12 primary schools)	BMI z-score^a^			
	N = 2184 (17%)	PA, NB	8.3 ± 2.2 yrs				
Sharma et al. (2016) [42]	Quasi-exp.	USA, 2013–2015	6 Intervention schools				Fruit intake (cups/ 1000 kcal/day)^c^
	16 weeks	16 weeks	6 Control schools				Vegetable intake (cups/1000 kcal/day)^c^
	N = 717 (26.6%)	NB	6.2 ± 0.4 yrs				Added sugar intake (tsp/1000 kcal/day)^c^
							Estimated percent of daily kilocalories from sugar beverages (%)^c^
							Fiber intake (grams/1000 kcal/day)^c^
							Fat intake (grams/ 1000 kcal/day) ^c^
							Average daily kilocalories (kcal/day) ^c^
							Whole grains intake (ounce/1000 kcal/day) ^c^
Siegrist et al. (2013) [30]	cluster RCT	Germany, 2006–2007	4 Intervention schools	BMI (kg/m^2^)^a^	Active ≥ 60 min (days/week)^b^		
	1 year	1 year	4 Control schools	SDS-BMI^a^			
	N = 826 (12%)	PA	8.4 ± 0.7 yrs				
Treu et al. (2015) [43]	Quasi-exp.	USA, 2010–2011	9 Intervention schools	BMI (kg/m^2^)^a^			
	6 months	6 months	9 Control schools	BMI z-score^a^			
	N = 1071 (*NR*)	PA, NB	8.7 ± 0.4 yrs				
Waters et al. (2018) [32]	Repeated cross-sectional	Australia, 2006–2009	12 Intervention schools	BMI (kg/m^2^)^a^	Active games at lunchtime (% children performing active games at lunchtime)^b^	TV viewing week day (% children watching TV 2 hours or less per week day)^c^	Serves of fruit^c^
	4–5 years	3.5 years	10 Control Schools	BMI z-score^a^	Being outside yesterday (% children being outside 2 hours or more after school yesterday)^c^	TV viewing weekend day (% children watching TV 2 hours or less per weekend day)^c^	Serves of vegetables^c^
	N = 3167 (N/A)	PA, NB	*NR*		Being outside weekend day (% children being outside 2 hours or more on a weekend day)^c^		Soft drink consumption (% children consuming any soft drink/day)^c^
							Fruit juice consumption (% children consuming any fruit juice/day)^c^
							Water consumption (% children consuming 2 or more glasses of water/day)^c^
Xu et al. (2015) [31]	RCT	China, 2010–2011	4 Intervention schools	BMI (kg/m^2^)^a^	Jogging/running frequency (% children with improved frequency)^b^	TV viewing or computer use (% children with reduced sedentary behavior)^b^	Red meat intake (%children with reduced intake)^b^
	10 months	10 months	4 Control schools		Walking frequency (% children with improved frequency)^b^		Fried snack intake (% children with reduced intake)^b^
	N = 1182 (6%)	PA, NB	10.2 ± 0.5 yrs		Ball playing (% children improved frequency)^b^		Soft drink consumption (% children with reduced intake)^b^
					Walking or riding bicycles to school (% children improved frequency)^b^		Vegetable intake (% children with increased intake)^b^
Xu et al. (2017) [47]	RCT	China, 2009–2010	21 Intervention schools	BMI (kg/m^2^)^a^			
	1 year	1 year	17 Control schools	BMI z-score^a^			
	N = 9867 (10.3%)	PA, NB	9.0 ± 0.5 yrs				

RCT = Randomized Controlled Trial, Quasi-exp. = Quasi-experimental, N/A = Not Applicable, *NR* = Not Reported, PA = Physical Activity, NB = Nutrition behavior, yrs = years, BMI = Body Mass Index, MVPA = Moderate-to-Vigorous intensity Physical Activity.

*Cong et al. (35) and Feng et al. (36) studied the effectiveness of the same intervention.

^a^ Measurement objectively assessed

^b^ Child-reported

^c^ Parent-reported

### Quality of the included studies

The methodological quality of eight studies (32%) was rated as weak [26, 32, 33, 36, 39–41, 46] (Table 3). Twelve studies (48%) were rated as being of moderate methodological quality [23, 24, 28, 30, 32, 35, 37, 38, 42–45] and five studies (20%) as being of high methodological quality [25, 27, 29, 31, 34]. Weak ratings were mainly due to information not being reported. For example, only four studies reported whether investigators were blinded to the intervention status of the participants [27, 32, 39, 42]. In addition, information on validity and reliability of data collection instruments or drop-out rates was missing in many studies [23, 26, 28, 30, 33, 35, 40, 41, 43, 46, 48].

**Table 3:** Quality rating of included studies (based on Thomas et al. (20)).

	**Selection bias**	**Study design**	**Confounders**	**Blinding**	**Data collection methods**	**Withdrawals and dropouts**	**Overall Score**
Alexander et al. (2014) [33]	Weak	Strong	Weak	Moderate	Weak	Moderate	**Weak**
Angelopoulos et al. (2009) [23]	Strong	Strong	Strong	Moderate	Strong	Weak	**Moderate**
Bacardí-Gascon et al. (2012) [44]	Weak	Strong	Strong	Moderate	Strong	Strong	**Moderate**
Bere et al (2006) [24]	Strong	Strong	Weak	Moderate	Strong	Moderate	**Moderate**
Cao et al. (2015) [25]	Strong	Strong	Strong	Moderate	Strong	Moderate	**Strong**
Centis et al. (2012) [34]	Strong	Strong	Strong	Moderate	Strong	Strong	**Strong**
Chomitz et al. (2010) [45]	Moderate	Moderate	Strong	Moderate	Strong	Weak	**Moderate**
Cong et al. (2012) [35]	Moderate	Strong	Strong	Moderate	Strong	Weak	**Moderate**
Crespo et al. (2012) [26]	Moderate	Strong	Strong	Moderate	Weak	Weak	**Weak**
Engelen et al. (2013) [27]	Strong	Strong	Strong	Moderate	Strong	Strong	**Strong**
Feng et al. (2016) [36]	Weak	Strong	Weak	Moderate	Strong	Strong	**Weak**
Hopper et al. (1996) [37]	Weak	Strong	Strong	Moderate	Strong	Strong	**Moderate**
Jiang et al. (2007) [28]	Strong	Strong	Strong	Moderate	Weak	Strong	**Moderate**
Kain et al. (2004) [38]	Weak	Strong	Strong	Moderate	Strong	Strong	**Moderate**
Li et al. (2014) [39]	Weak	Strong	Weak	Strong	Strong	Strong	**Weak**
Manios et al. (1999) [29]	Strong	Strong	Strong	Moderate	Strong	Strong	**Strong**
Muller et al. (2001) [46]	Weak	Weak	Weak	Moderate	Weak	Weak	**Weak**
Prelip et al. (2012) [40]	Weak	Strong	Strong	Moderate	Strong	Weak	**Weak**
Sanigorski et al. (2013) [41]	Weak	Strong	Moderate	Moderate	Weak	Strong	**Weak**
Sharma et al. (2016) [42]	Weak	Strong	Strong	Moderate	Moderate	Moderate	**Moderate**
Siegrist et al. (2013) [30]	Strong	Strong	Strong	Moderate	Weak	Strong	**Moderate**
Treu et al. (2015) [43]	Moderate	Strong	Strong	Moderate	Strong	Weak	**Moderate**
Waters et al. (2018) [32]	Weak	Moderate	Weak	Moderate	Weak	Moderate	**Weak**
Xu et al. (2015) [31]	Strong	Strong	Strong	Moderate	Strong	Strong	**Strong**
Xu et al. (2017) [47]	Weak	Strong	Strong	Moderate	Strong	Strong	**Moderate**

### Intervention results regarding BMI or BMI z-score

Eleven of the eighteen studies measuring intervention effects on BMI or BMI z-score found favorable results [23, 25, 28, 29, 33, 34, 38, 39, 41, 45, 47]. Of these, seven studies were positively effective on BMI and BMI z-score [28, 29, 34, 39, 41, 45, 47] and four studies found mixed results [23, 25, 33, 38]. Of the studies with mixed results, one study found the intervention to be positively effective regarding BMI, but ineffective regarding the BMI z-score [23], the remaining three studies found effective results for particular subgroups: normal and overweight subgroups [33]; children who were overweight and obese at baseline [25]; and boys [38] (S2 Table). Of the studies with favorable results, effect sizes for BMI or BMI z-score were mainly small (ES -0.04 to -0.27) [25, 34, 38, 39, 41, 45]. Two studies had a moderate effect size (ES -0.34 and -0.48) [23, 29] and one study found a large effect on BMI (ES -0.79) [28] (S2 Table). The study quality of these studies was strong for three studies [25, 29, 34], moderate for five studies [23, 28, 38, 45, 47] and weak for three studies [33, 39, 41]. Six studies reported their intervention to be ineffective regarding BMI and BMI z-score [26, 30–32, 36, 43], although three of these showed a positive trend (ES -0.10 and -0.01, respectively) [31, 32, 36]. One study found negative results [44]. This was a pretest-posttest study without a comparison group.

### Intervention results regarding physical activity behavior

Except for two studies, all eleven studies on PA found favorable results on at least one PA outcome measure [23, 26, 27, 29, 31, 34, 39, 44, 46]: four studies found significant positive results for all PA outcome measures [29, 34, 39, 46] and five studies found significant positive results for at least one PA outcome but were ineffective regarding other PA outcomes [26, 27, 31, 34, 44]. Small to large significant positive effects were found on total daily moderate-to-vigorous PA (MVPA) (ES 0.41 and 0.48) [23, 39], MVPA during school break time (ES 0.19) [27], leisure-time MVPA (ES 0.98) [29], time spent in outdoor activities (ES 0.49) [34], daily physical activities [46], frequency of jogging/running [31], number of sports participated in during the past year (ES 0.10) [26], and supervised sports or dancing per week (ES 0.34) [44]. PA was mostly self-reported by the children [23, 31, 39, 44, 46] or the parents [26, 29, 34]. One study measured PA by the use of accelerometers [27]. The methodological study quality of the studies with favorable results was strong for four studies [27, 29, 31, 34], moderate for two studies [23, 44] and weak for three studies [26, 39, 46]. Two studies found the intervention to be ineffective on PA [30, 32], however one study found a positive trend (ES 0.20) [30].

### Intervention results regarding sedentary behavior

Six of the eight studies measuring SB found favorable results [27, 31, 34, 35, 44, 46]. Of these, four studies reported merely significant positive results [31, 34, 35, 46] and two reported significant positive result for at least one SB outcome but were ineffective regarding other SB outcomes [27, 44]. Small to moderate significant positive effects were found on TV watching per week (ES -0.38) [34] and TV watching per day (ES -0.15) [44, 46], screen-time behavior [35], TV viewing and computer use [31], sitting per day (ES -0.20) [44] and SB during school break time (defined by the use of accelerometer-specific cut-off points) (ES -0.02) [27]. SB was mainly measured via child-questionnaire [31, 44, 46] or parent-questionnaire [34, 35]. One study used accelerometers to measure SB [27]. Three studies were of strong methodological quality [27, 31, 34], two of moderate [35, 44] and one of weak quality [46]. Two interventions were found to be ineffective regarding frequency TV viewing. Although not significant, the results were in favor of the intervention group (ES -0.41) [26, 32].

### Intervention results regarding nutrition behavior

Twelve studies measured intervention effects on NB. Five studies found favorable results regarding NB outcomes measured [31, 36, 37, 42, 46], of which two studies reported merely statistically significant positive results for all NB outcomes [36, 46] and three studies reported significant positive results, but were ineffective regarding other NB outcomes [31, 37, 41]. Small to moderate significant positive effects were seen on daily sugar-sweetened beverages consumption (ES -0.42) [36], fruit and vegetable consumption (ES 0.17, 0.21, 0.35) [37, 42, 46], added sugar intake (ES -0.21) [42], and red meat consumption [31]. The methodological study quality was mainly weak [36, 41, 46]; two studies were of moderate [37] and strong [31] methodological quality. Two studies reported mixed results: with statistically significant positive results for some NB outcomes and statistically negative results for other outcomes [23, 44]. Five studies found the intervention to be ineffective on NB [24, 26, 29, 32, 40].

### Intervention characteristics

The intervention durations varied from ten weeks [37] to four years [46] (Table 2). It seems that interventions of longer duration (at least one year) were more likely to lead to favorable results regarding weight status [23, 25, 28, 29, 41, 45, 47], but not for PA, SB or NB. Two interventions targeted PA only [27, 30] and three interventions targeted NB only [24, 40, 42]. These interventions were mainly ineffective. The remaining interventions targeted children’s PA as well as their NB. Eight studies reported to target children’s SB (e.g. reduce TV viewing) [26, 30, 35, 36, 39, 41, 44–46].

#### The child’s socio-cognitive determinants targeted

Five socio-cognitive determinants of the children targeted by the interventions could be distinguished: knowledge, awareness, attitude, self-efficacy and intrinsic motivation (Table 4). Except for three studies [27, 32, 34], all interventions educated the children on nutrition, physical activity, or health with the aim of increasing knowledge about EBRBs. Active ways of nutrition education (cooking classes) and physical education (in the form of extra and better-quality physical education) were implemented by 14 interventions, aiming to increase energy balance-related skills and self-efficacy [23, 24, 26, 29–37, 43–45]. Seven interventions aimed to increase the children’s awareness on their own PA or NB, by asking them to monitor their behavior [23, 24, 30, 31, 34, 39, 44]. Six studies emphasized that their intervention was fun/enjoyable for the children, in order to increase intrinsic motivational regulation [23, 27, 29–31, 34]. Four interventions aimed to change children’s attitudes toward PA and/or nutrition [23, 24, 34, 40]. No pattern was found between the child’s socio-cognitive determinants targeted and intervention effectiveness.

**Table 4:** Children’s socio-cognitive determinants, the community, school and family environmental types targeted, and effectiveness of the interventions.

	Socio-cognitive determinants					Environment												Effectiveness‡			
	Child					Community				School				Family							
Authors	Kn	Aw	At	S-E	I-M	Ph	So	Ec	Po	Ph	So	Ec	Pol	Ph	So	Ec	Po	BMI (z)	PA	SB	NB
Alexander et al. (2014) [33]	**•**			**•**							**•**		**•**		**•**		**•**	**+/0**			
Angelopoulos et al. (2009) [23]	**•**	**•**	**•**	**•**	**•**					**•**	**•**		**•**	**•**	**•**			**+/0**	**+**		**+/-/0**
*Bacardí-Gascon et al*. *(2012)† [44]*	**•**	**•**		**•**						**•**	**•**				**•**			**-**	**+/0**	**+/0**	**+/-/0**
Bere et al. (2006) [24]	**•**	**•**	**•**	**•**						**•**	**•**	**•**		**•**	**•**		**•**				**0**
Cao et al. (2015) [25]	**•**									**•**	**•**		**•**		**•**		**•**	**+/0**			
Centis et al. (2012) [34]		**•**	**•**	**•**	**•**					**•**	**•**			**•**	**•**			**+**	**+/0**	**+**	
*Chomitz et al*. *(2010)† [45]*	**•**			**•**			**•**		**•**	**•**	**•**		**•**		**•**			**+**			
Cong et al. (2012) [35] / Feng et al. (2016) [36]	**•**			**•**						**•**	**•**			**•**	**•**		**•**	**0**		**+**	**+**
Crespo et al. (2012) [26]	**•**			**•**		**•**	**•**		**•**	**•**	**•**		**•**	**•**	**•**		**•**	**0**	**+/0**	**0**	**0**
Engelen et al. (2013) [27]					**•**					**•**	**•**				**•**				**+/0**	**+/0**	
Hopper et al. (1996) [37]	**•**			**•**							**•**				**•**	**•**					**+/0**
Jiang et al. (2007) [28]	**•**										**•**			**•**	**•**		**•**	**+**			
Kain et al. (2004) [38]	**•**									**•**	**•**	**•**			**•**			**+/0**			
Li et al. (2014) [39]	**•**	**•**									**•**		**•**	**•**	**•**			**+**	**+**		
Manios et al. (1999) [29]	**•**			**•**	**•**						**•**				**•**			**+**	**+**		**0**
Müller et al. (2001) [46]	**•**										**•**			**•**	**•**		**•**		**+**	**+**	**+**
Prelip et al. (2012) [40]	**•**		**•**							**•**	**•**			**•**	**•**		**•**				**0**
Sanigorski et al. (2008) [41]	**•**					**•**	**•**			**•**	**•**	**•**	**•**		**•**		**•**	**+**			
Sharma et al. (2016) [42]	**•**										**•**			**•**	**•**	**•**	**•**				**+/0**
Siegrist et al. (2013) [30]	**•**	**•**		**•**	**•**					**•**	**•**		**•**		**•**		**•**	**0**	**0**		
Treu et al. (2015) [43]	**•**			**•**							**•**			**•**	**•**	**•**		**0**			
Waters et al. (2018) [32]				**•**						**•**	**•**	**•**	**•**		**•**			**0**	**0**	**0**	**0**
Xu et al. (2015) [31]	**•**	**•**		**•**	**•**					**•**	**•**		**•**		**•**		**•**	**0**	**+/0**	**+**	**+/0**
Xu et al. (2017) [47]	**•**									**•**	**•**				**•**			**+**			

Kn = Knowledge, Aw = Awareness, At = Attitude, S-E = Self-efficacy, I-M = Intrinsic Motivation, Ph = Physical, So = Sociocultural, Ec = Economic, Po = Political, BMI (z) = Body Mass Index or Body Mass Index z-score, PA = Physical Activity Behavior, SB = Sedentary Behavior, NB = Nutrition Behavior.

† Effect sizes are changes over time (no control group). Other effect sizes are effect sizes for the standardized mean difference (end line—baseline) between intervention group and control group.

‡ The effectiveness of the studies is presented as positive (+): all results for the particular outcome were statistically significant in favor of the intervention group

Mixed effects (+/-; +/0; +/-/0): at least one result was statistically significant in favor of the intervention group, whereas the other results were not

Negative effects (-): all results for the particular outcome were statistically significant in favor of the control group

Ineffective (0): no statistically significant results for one of the groups.

#### Environmental types targeted

With the exception of the intervention by Manios et al. [29], all interventions targeted at least three environmental types in the school and family environment combined (Table 4). All interventions targeted at least the social school environment (teachers and/or other school staff) and the social family environment (the parents). Sixteen interventions aimed to change the physical school environment [23–27, 30–32, 34–36, 38, 40, 41, 44, 45, 47]. Physical changes to the school environment included changes to the school menu, gymnasium equipment and school playground improvement. The third most targeted environmental type was the political school environment (n = 10) (e.g. school health policies) and the political family environment (n = 11); parents were counseled on implementing parental rules which stimulate healthy EBRBs at home [24–26, 28, 30, 31, 33, 35, 36, 40–42, 46]. Four studies considered their interventions to be community-based [26, 36, 41, 45]. They targeted two or three environmental types in the community environment, one of which was the social community environment: training professionals of afterschool organizations [45], community health workers [26, 36], and club coaches and canteen staff [41]. There was no consistent pattern to the results in terms of the types and number of environmental types targeted.

#### Parental involvement components

All interventions involved the parents *directly*; 17 interventions additionally applied *indirect* involvement strategies by providing written information to parents, like newsletters, brochures, information sheets, recipe cards and lists of tips (n = 13) [24–26, 28–32, 35, 36, 41–43, 47], and/or by requesting parents to assist their child with the intervention-related homework (n = 8) [23, 25, 26, 29, 30, 37, 42, 43] (Table 5). Parents were predominantly *directly* involved in the intervention by attending educational sessions (n = 17) [23–25, 27–32, 34, 38–40, 42, 44, 46, 47]. These sessions were mainly organized as group sessions for the parents [24, 27, 28, 31, 34, 38–40, 44, 46]. Eight interventions implemented energy balance-related activities for parents and children (family activities), e.g. family cooking nights, fruit and vegetable bazaars, activities in the supermarket [23, 24, 33, 35, 36, 41–43, 45]. In seven interventions one-on-one counseling was provided, mostly by home visits [26, 33, 35, 36, 46], or telephone counseling [34, 37]. One study did not report the counseling method used [45]. Five interventions additionally targeted parents of overweight children [28, 33, 35, 36, 45, 46]; in these interventions parental counseling sessions were held [33, 35, 36, 45, 46] or information meetings were arranged [28]. These interventions were effective on children’s BMI [28, 33], BMI z-score [45], PA [46], SB and NB [35, 36, 46]. In the four interventions in which parents were provided a report on their child’s health status [23, 29, 44, 45] favorable results were found regarding the intervention effect on children’s BMI [23, 29] and BMI z-score [45], their PA [29, 44] and SB [44].

**Table 5:** Parental involvement components of interventions and effectiveness of the interventions.

	**Direct parental involvement**			**Indirect parental involvement**			**Effectivity**‡			
**Authors**	Educational sessions	Family activities	One-one-one parent counseling	Support with child’s homework	Provision of written information	Report with health status child	BMI z / BMI	PA	SB	NB
Alexander et al. (2014) [33]		**•**	**•**				**+/0**			
Angelopoulos et al. (2009) [23]	**•**	**•**		**•**		**•**	**+/0**	**+**		**+/-/0**
*Bacardí-Gascon et al*. *(2012)† [44]*	**•**					**•**	**-**	**+/0**	**+/0**	**+/-/0**
Bere et al. (2006) [24]	**•**	**•**			**•**					**0**
Cao et al. (2015) [25]	**•**			**•**	**•**		**+/0**			
Centis et al. (2012) [34]	**•**		**•**				**+**	**+/0**	**+**	
*Chomitz et al*. *(2010)† [45]*		**•**	**•**			**•**	**+**			
Cong et al. (2012) [35] / Feng et al. (2016) [36]		**•**	**•**		**•**		**0**		**+**	**+**
Crespo et al. (2012) [26]			**•**	**•**	**•**		**0**	**+/0**	**0**	**0**
Engelen et al. (2013) [27]	**•**							**+/0**	**+/0**	
Hopper et al. (1996) [37]			**•**	**•**						**+/0**
Jiang et al. (2007) [28]	**•**				**•**		**+**			
Kain et al. (2004) [38]	**•**						**+/0**			
Li et al. (2014) [39]	**•**						**+**	**+**		
Manios et al. (1999) [29]	**•**			**•**	**•**	**•**	**+**	**+**		**0**
Müller et al. (2001) [46]	**•**		**•**					**+**	**+**	**+**
Prelip et al. (2012) [40]	**•**									**0**
Sanigorski et al. (2008) [41]		**•**			**•**		**+**			
Sharma et al. (2016) [42]	**•**	**•**		**•**	**•**					**+/0**
Siegrist et al. (2013) [30]	**•**			**•**	**•**		**0**	**0**		
Treu et al. (2015) [43]		**•**		**•**	**•**		**0**			
Waters et al. (2018) [32]	**•**				**•**		**0**	**0**	**0**	**0**
Xu et al. (2015) [31]	**•**				**•**		**0**	**+/0**	**+**	**+/0**
Xu et al. (2017) [47]	**•**				**•**		**+**			

BMI (z) = Body Mass Index or Body Mass Index z-score, PA = Physical Activity Behavior, SB = Sedentary Behavior, NB = Nutrition Behavior.

† Effect sizes are changes over time (no control group). Other effect sizes are effect sizes for the standardized mean difference (end line—baseline) between intervention group and control group.

‡ The effectiveness of the studies is presented as positive (+): all results for the particular outcome were statistically significant in favor of the intervention group

Mixed effects (+/-; +/0; +/-/0): at least one result was statistically significant in favor of the intervention group, whereas the other results were not

Negative effects (-): all results for the particular outcome were statistically significant in favor of the control group

Ineffective (0): no statistically significant results for one of the groups.

## Discussion

The aims of this systematic literature review were to explore the effectiveness of school-based physical activity and nutrition interventions with direct parental involvement regarding children’s BMI, BMI z-score and their EBRBs and to distinguish the children’s socio-cognitive determinants and environmental types targeted in these interventions. A total of 25 studies describing 24 school-based interventions with direct parental involvement were included. The majority of the studies reporting results regarding BMI and BMI z-score (11 of 18 studies) found favorable, though mainly small, effects. In addition, almost all studies that measured effects on physical activity behavior (9 of 11 studies) or sedentary behavior (6 of 8 studies) showed favorable results. The effects on nutrition behavior were inconclusive.

The results of this systematic review show more beneficial results for physical activity behavior and sedentary behavior compared to previous systematic reviews conducted on the effectiveness of these types of interventions [12, 13]. A possible explanation for the discrepancy in the results may be the fact that this study included only school-based interventions in which parents were directly involved, while other studies included mainly interventions with indirect parental involvement. This may indicate the importance of directly engaging parents in school-based interventions that aim to improve children’s EBRBs instead of using indirect strategies such as intervention-related newsletters.

While no consistent pattern was found between intervention’s effectiveness and the number of environmental types and specific environmental types targeted, a successful interaction between the social and physical environment in the school and the family environment is presumably important for school-based interventions to be effective. Most studies targeted both the social and the physical environment in the school and the family environment. Research has confirmed the influence of both the physical and social environment in the school and the family environmental setting on children’s EBRBs [49–52] and the enhancing effect that occurs when the social and physical environments interact [18]. This enhancing effect is seen in former studies in both the school setting [52] and the home setting [53]. The lack of a consistent pattern between targeted determinants and study outcomes may also be explained by the focus on more distal outcomes (BMI, EBRBs) used in this study. The mediating role of these targeted determinants on the outcomes fell out of scope for this review. However, it might be important to study intervention effects on these determinants, as this may be part of the explanation of effectiveness on BMI and EBRBs. In addition, the current review did not take into account the behavioral change techniques (BCTs) used in the intervention studies. There was substantial missing information across studies regarding BCTs, limiting comparability of the study results. However, taking into account BCTs may also be an important factor in understanding intervention effectiveness.

The results of this review should be interpreted with caution: the methodological quality between the studies varied greatly. In particular, the results regarding physical activity and sedentary behavior should be interpreted carefully, as most PA and SB were measured by self-reporting. Only one study objectively assessed PA and SB with accelerometers [27]. Subjective measurements are prone to social desirability and recall bias [54]. However, the overall methodological quality of the studies did not apparently influence the results on PA and SB, as both studies of weak and strong quality had comparable results. This is something also encountered by other researchers, showing less strong effects on robust outcome measures (i.e. BMI or PA) for studies rated with a strong study design compared to studies with a weak study design [55]. Furthermore, process-related quality measures such as fidelity or compatibility, may be also important factors related to effectiveness in addition to research design aspects. This could be an aspect to consider in future reviews when assessing the quality of the included studies.

An explanation for the inconclusive results regarding intervention effectiveness on nutrition behavior might be intervention duration. Nutrition behavior is complex and it takes time to change dietary habits [56], thus it may be likely that interventions of longer duration will be more effective in changing dietary behaviors. However, this possible association between intervention duration and nutrition behavior outcomes has not yet been explored as the majority of the studies were of relative short duration (one year or less). This emphasizes the need to conduct long-term school-based interventions measuring effects on nutrition behavior. The need for long-term interventions when aiming to change children’s weight status and EBRBs is confirmed by the studies measuring intervention effects on BMI or BMI z-score: long-term interventions (at least one year) were more likely to have favorable effects on children’s weight status. In addition, interventions should target more than one EBRB. Interventions targeting more than one EBRB were more likely to be effective than interventions targeting a single EBRB. This result is in line with the empirical evidence that these behaviors tend to cluster, e.g. a clustering of high sedentary behavior and high levels of physical activity, indicating that a healthy single behavior not necessarily results in an overall healthy lifestyle [57]. Therefore, limiting interventions to a single behavior may result in missing an essential component of the energy balance, which may lead to less favorable results in relation to child outcomes [57].

Paying additional attention to the parents of an overweight or obese child may also be important for intervention effectiveness on children’s weight status and EBRBs. One risk of school-based interventions is that healthy children may benefit more from the interventions than high-risk children [58]. Additional interventions or more intensive interventions for high-risk populations may overcome this problem [59, 60]. All interventions in which the parents of high-risk children were additionally targeted were effective at improving BMI, BMI z or EBRBs.

The implementation of school-based interventions with direct parental involvement is challenging since achieving parental engagement in school-based interventions is considered difficult [61]. Involving parents directly is even more challenging. The large number of studies on school-based interventions with indirect parental involvement [62] compared to the low number of studies on school-based interventions with direct parental involvement, confirms this assumption. There is a clear need to better operationalize parental involvement in school-based interventions in order to increase parental engagement. Perhaps parental involvement should be the primary focus of these types of interventions, taking into account parental perspectives and parental needs at first, and secondarily focusing on schools and children [63]. A focus on interpersonal aspects, such as parent-child bonding or providing set family time, which were rated by parents equally important as health reasons, may help in convincing them to participate in intervention activities [64]. The ‘Healthy Dads, Healthy Kids’-intervention is an example of a successful intervention, both on outcomes and retaining participation, incorporating these aspects in their intervention program [65].

A qualitative study on engaging families in physical activity research found that parents were more willing to engage in interventions when they received information about their children’s health [66]. Two intervention studies that organized sessions in which the children’s health status reports were distributed to the parents and information was provided about health-promoting strategies, showed high levels of parental attendance at these sessions [23, 29]. Lastly, a study among parents and early childhood professionals showed a preference for internet-delivered interventions in order increase parental engagement [67]. Evidence regarding effective strategies to involve parents in school-based interventions is lacking [61]. These results need to be confirmed by future research, since information on parental attendance at information sessions was lacking in most papers.

### Limitations of the studies

There are some limitations of the studies included in this review. Methodological study quality was difficult to rate in most papers because of a lack of detail. As a result, it might be underestimated. A second limitation was the great variation in outcome measures of EBRBs. We tried to overcome this problem by calculating effect sizes. Since only one author answered our request for additional information to allow the calculation of effect sizes, it was impossible to calculate the effect sizes for all studies. Another limitation was the incomplete description of most interventions. This limitation impeded comparison and extraction of information and may have biased the results as presented in this systematic review.

### Strengths and limitations of the systematic review

To our knowledge this is the first systematic review aiming to disentangle the socio-cognitive determinants and different environmental types targeted in the school and home environment to explain the intervention effectiveness of school-based physical activity and nutrition interventions with direct parental involvement. We used the EnRG framework [17], which has been employed in other studies to analyze intervention content (e.g. [68]). Methodological strengths of this systematic review were the use of the EPHPP tool to assess the methodological quality of the studies (this quality assessment tool has proven content and construct validity) [20]; the use of the PRISMA statement [19] and the calculation of effect sizes (Cohen’s d).

The limitations of this systematic review should also be acknowledged. There is a risk of publication bias, as we used only four databases and included only articles written in English. Another limitation may be the inclusion of studies with a weak methodology. In most cases, the weak methodological ratings were due to missing information. We decided to include these studies anyway, as we did not know whether the components determining quality were indeed not implemented by the researchers or whether they merely failed to report the information. The inclusion of studies with any other design than a randomized controlled trial (RCT) can also be a limitation of this study, since RCTs are considered the gold standard [69]. However, the results of quasi-experimental study designs are valuable because of their external validity, and that study design is considered more appropriate for these types of interventions [70].

### Recommendations

This systematic review demonstrates the potential of school-based interventions with direct parental involvement to improve BMI, BMI z, and physical activity and decrease sedentary behavior. We recommend that policymakers and practitioners develop and implement school-based interventions with direct parental involvement, focus on multiple EBRBs simultaneously to take into account the total energy balance, and target different environmental types, in particular the social and physical environments, both within the school and the home. We recommend that sustainability of interventions should be carefully considered as sustainable intervention (twelve months or longer) appear to be more effective compared to studies of shorter duration. This may require a shift in focus and budgeting, implementing less but more extensive intervention activities. To enable the implementation of these interventions, research should focus on effective strategies to engage parents and enhance parental involvement. This may need a shift in focus from primarily focusing on schools and children towards parents, making this the key element and taking into account their needs and perspectives. Further, we recommend an extended exploration of the role of behavioral change techniques alongside the types of environments and socio-cognitive determinants. This may add to the ability to explain intervention effectiveness, however fell out of scope for the current review. In addition, we recommend future studies to study the effectiveness on intermediate outcomes (e.g. socio-cognitive and environmental determinants) in order to explore the pathways of effectiveness of these types of interventions.

## Supporting information

S1 Table Search strategy Pubmed. (DOCX)

S2 Table Intervention effects on outcome measures. (DOCX)

S1 File PRISMA statement. (DOCX)
