# Supplementary material for: Effectiveness of school-based physical activity and nutrition interventions with direct parental involvement on children’s BMI and energy balance-related behaviors – A systematic review
Source: PLoS One. 2018 Sep 27;13(9):e0204560. doi: 10.1371/journal.pone.0204560 (PMC6160096; doi:10.1371/journal.pone.0204560)
Supplement: S1 Table — (DOCX) [file pone.0204560.s001.docx]

**Supplemental file S1 Table**

S1 Table. Search strategy Pubmed.

| **Category** | **Search terms** |
| --- | --- |
| Child | (Child [Mesh] OR Child [Title/Abstract]) OR Children [Title/Abstract] OR (Child, preschool [Mesh] OR Child, preschool [Title/Abstract]) OR Children, preschool [Title/Abstract] OR Minors [Mesh] OR Minor [Title/Abstract] OR Minors [Title/Abstract] OR (Pre-schoolers [Title/Abstract] OR Preschoolers [Title/Abstract]) OR Preschooler [Title/Abstract] OR Toddler [Title/Abstract] OR Toddlers [Title/Abstract] OR (Infant [Mesh] OR Infant [Title/Abstract]) OR Infants [Title/Abstract]) |
| Intervention components | ((“Motor activity” [Mesh] OR physical activity [Title/Abstract] OR physical activities [Title/Abstract]) OR (“Life Style” [Mesh] OR “lifestyle” [Title/Abstract] OR lifestyle [Title/Abstract] OR lifestyles [Title/Abstract]) OR “Energy balance” [Title/Abstract] OR (“Diet, Food and Nutrition” [Mesh] OR Food [Mesh] OR “healthy food” [Title/Abstract] OR “unhealthy food” [Title/Abstract] OR (Diet [Mesh] OR Diet [Title/Abstract] OR Dietary [Title/Abstract] OR Diets [Title/Abstract]) OR Nutrition [Title/Abstract] OR “Child Nutrition” [Mesh] OR (“healthy eating” [Title/Abstract] OR “unhealthy eating” [Title/Abstract]) OR (“energy intake” [Mesh] OR “energy intake” [Title/Abstract]) OR (“Sedentary Life Style” [Mesh] OR “Sedentary Life Style”[Title/Abstract] OR “Sedentary behavior” [Title/Abstract] OR “Sedentary behaviour” [Title/Abstract]) |
| Pre-School/School | (“Schools, nursery” [Mesh] OR (“Child Day Care Centers” [Mesh] OR “Day Care” [Title/Abstract] OR “Daycare” [Title/Abstract] “Day Cares” [Title/Abstract] OR “Daycares” [Title/Abstract]) OR (Pre-school [Title/Abstract] OR Pre-schools [Title/Abstract] OR Preschool [Title/Abstract] OR Preschools [Title/Abstract]) OR (Kindergarten [Title/Abstract] OR Kindergartens [Title/Abstract]) OR (Nursery [Title/Abstract] OR Nurseries [Title/Abstract]) OR (Playgroup [Title/Abstract] OR Playgroups [Title/Abstract]) OR (Schools [Mesh] OR School [Title/Abstract] OR Schools [Title/Abstract] OR “Primary school” [Title/Abstract] OR “Primary schools” [Title/Abstract]) OR (“School based” [Title/Abstract] OR “School centered” [Title/Abstract])) |
| Family | (Parents [Mesh] OR Parent [Title/Abstract] OR Parents [Title/Abstract]) OR (Fathers [Mesh] OR Fathers [Title/Abstract] OR Father [Title/Abstract]) OR (Mothers [Mesh] OR Mothers [Title/Abstract] OR Mother [Title/Abstract]) OR (Caregiver [Title/Abstract] OR Caregivers [Title/Abstract]) OR (Family [Mesh] OR Family [Title/Abstract] OR Families [Title/Abstract]) OR (“Family based” [Title/Abstract] OR Home [Title/Abstract] OR “Home based” [Title/Abstract]) OR Parental [Title/Abstract]) |
| Intervention | (Intervention [Title/Abstract] OR Interventions [Title/Abstract]) |
| Effectiveness | ((Evaluation [Title/Abstract] OR evaluations [Title/Abstract]) OR (“evaluation studies” [Publication type] OR “evaluation studies as topic” [Mesh]) OR (effects [Title/Abstract] OR effectiveness [Title/Abstract] OR effectivity [Title/Abstract] OR effective [Title/Abstract] OR effect [Title/Abstract]) OR “pre post test” [Title/Abstract] OR “pre post tests” [Title/Abstract]) |
