# Supplementary material for: Effectiveness of school-based physical activity and nutrition interventions with direct parental involvement on children’s BMI and energy balance-related behaviors – A systematic review
Source: PLoS One. 2018 Sep 27;13(9):e0204560. doi: 10.1371/journal.pone.0204560 (PMC6160096; doi:10.1371/journal.pone.0204560)
Supplement: S2 Table — (DOCX) [file pone.0204560.s002.docx]

**Supplemental file S2**

**S2 Table. Intervention effects on BMI/BMI z-score, physical activity behavior, sedentary behavior and nutrition behavior.**

| **Authors** | **BMI/BMI z-score results** | **Physical activity behavior results** | **Sedentary behavior results** | **Nutrition behavior results** |
| --- | --- | --- | --- | --- |
| Alexander et al. (2014) [33] | *BMI percentile <25:*  A larger decrease in BMI for the intervention group (0.090 kg/m^2^) compared to the control group (0.029 kg/m^2^) (p = 0.64) | NA | NA | NA |
|  | *BMI percentile 25 to <50:*  A decrease in BMI for the intervention group (0.161 kg/m^2^) and an increase for the control group (0.133 kg/m^2^) (p = 0.027) |  |  |  |
|  | *BMI percentile 50 to <75:*  A decrease in BMI for the intervention group (0.141 kg/m^2^) and an increase for the control group (0.109 kg/m^2^) (p = 0.045) |  |  |  |
|  | *BMI percentile 75 to <95:*  A smaller increase in BMI for the intervention group (0.074 kg/m^2^) compared to the control group (0.684 kg/m^2^) (p = 0.00007) |  |  |  |
|  | *BMI percentile >95*:  A smaller increase in BMI for the intervention group (0.48 kg/ m^2^) compared to the control group (0.78 kg/ m^2^) (p = 0.085) |  |  |  |
| Angelopoulos et al. (2009) [23] | A larger decrease in BMI z-score for the intervention group (-0.46) compared to the control group (-0.16) (p = 0.074) **ES -0.33** | An increase in MVPA for the intervention group (2.2 min/day) and a decrease for the control group (-16.4 min/day) (p = 0.041)  **ES 0.48** | NA | An increase of fruit exchanges/day for the intervention group (0.4 exchanges/day) and a decrease of for the control group (-0.2 exchanges/day) (p = 0.044) **ES 0.44** |
|  | A decrease in BMI for the intervention group (-1.1 kg/m^2^) compared to an increase in BMI for the control group (0.1 kg/m^2^) (p = 0.047) **ES -0.34** |  |  | A decrease in vegetables exchanges/day for the intervention group (-0.02 exchanges/day) and no change for the control group (0.0 exchanges/day) (p = 0.680) **ES -0.29** |
|  |  |  |  | A decrease in dairy intake for the intervention group (-0.2 exchanges/day) compared to an increase for the control group (0.2 exchanges/day) (p= 0.008)  **ES -0.30** |

**S2 Table. Intervention effects on BMI/BMI z-score, physical activity behavior, sedentary behavior and nutrition behavior (continued).**

| **Authors** | **BMI/BMI z-score results** | **Physical activity behavior results** | **Sedentary behavior results** | **Nutrition behavior results** |
| --- | --- | --- | --- | --- |
| Angelopoulos et al. (2009) [23] |  |  |  | A larger decrease in meat intake for the intervention group (-0.8 exchanges/day) compared to the control group (-0.7 exchanges/day) (p = 0.065) **ES -0.03** |
|  |  |  |  | A decrease in grains intake for the intervention group (-0.9 exchanges/day) compared to an increase for the control group (0.3 exchanges/day) (p = 0.055)  **ES -0.38** |
|  |  |  |  | A decrease in fats and oils intake for the intervention group (-1.6 exchanges/day) and an increase for the control group (0.7 exchanges/day) (p = 0.028) **ES -0.47** |
|  |  |  |  | A decrease in sweets and beverage intake for the intervention group (-0.8 exchanges/day) and an increase for the control group (0.2 exchanges/day) (p = 0.039) **ES -0.41** |
| Bacardí-Gascon et al. (2012) [44] | An increase in BMI z-score for the intervention group (0.09) (no control group) (p = 0.001) **ES 0.07** | A decrease in outdoors play for the intervention group (-0.03 h/day) (no control group) (p = 0.63) **ES -0.03** | A decrease in hours sitting per day for the intervention group (-0.49 h/day) (no control group) (p = 0.001) **ES -0.20** | A decrease in fruit portions per weekday for the intervention group (-0.08 portions/weekday) (no control group) (p = 0.26) **ES -0.07** |
|  | An increase in BMI for the intervention group (1.3 kg/m^2^) (no control group) (p = 0.001) **ES 0.33** | An increase in physical education for the intervention group (0.07 h/week) (p = 0.003) **ES 0.23** | A decrease in TV watching for the intervention group (-0.16 h/day) compared to the control group (p = 0.02) **ES -0.15** | An increase in vegetable portions per weekday for the intervention group (0.09 portions/weekday) (no control group) (p = 0.007) **ES 0.15** |
|  |  | An increase supervised sports or dance for the intervention group (0.76 h/week) (no control group) (p = 0.0001) **ES 0.34** | An increase in computer and video games usage for the intervention group (0.07 h/day) (p = 0.31) **ES 0.07** | An increase in sugar sweetened beverages consumption for the intervention group (0.62 portions/day) (no control group) (p = 0.0001) **ES 0.67** |
|  |  |  |  | An increase in soda consumption (0.01 portions/day) (no control group) (p = 0.56)  **ES 0.02** |
|  |  |  |  | A decrease in chocolate and candy intake (0.06 portions/day) (no control group) (p = 0.24)  **ES -0.06** |
| Bacardí-Gascon et al. (2012) |  |  |  | A decrease in snack intake (0.08 portions/day) (no control group) (p = 0.03)  **ES -0.10** |

**S2 Table. Intervention effects on BMI/BMI z-score, physical activity behavior, sedentary behavior and nutrition behavior (continued).**

| **Authors** | **BMI/BMI z-score results** | **Physical activity behavior results** | **Sedentary behavior results** | **Nutrition behavior results** |
| --- | --- | --- | --- | --- |
| Bere et al. (2006) [24] | NA | NA | NA | *After 8 months (end of intervention):*  A larger decrease in portions fruit and vegetable per day at school for the intervention group (-0.07 portions/day) compared to the control group (-0.02 portions/day) (p = 0.53) **ES -0.04** |
|  |  |  |  | A larger decrease in portions fruit and vegetable per day all day for the intervention group (-0.62 portions/day) compared to the control group (-0.44 portions/day) (p = 0.23) **ES -0.04** |
|  |  |  |  | *After 1 year and 8 months (follow-up):*  A decrease in portions fruit and vegetable per day at school for the intervention group (-0.22 portions/day) and a small increase for the control group (from 0.02 portions/day) (p = 0.76) **ES -0.19** |
|  |  |  |  | A larger decrease in portions fruit and vegetable per day all day for the intervention group (-0.88 portions/day) compared to the control group (-0.42 portions/day) (p = 0.41) **ES -0.36** |
| Cao et al. (2015) [25] | *Normal weight at baseline:*  No difference in BMI z-score for the intervention group compared to the control group (β = 0.002) (p = 0.638) | NA | NA | NA |
|  | *Overweight at baseline:*  A smaller increase in BMI z-score for the intervention group compared to the control group (β = -0.030) (p = 0.002) |  |  |  |
|  | *Obesity at baseline:*  A smaller increase in BM z-score for the intervention compared to the control group (β = -0.046) (p < 0.001) |  |  |  |
|  | *Total group:*  A smaller increase in BMI z-score for the intervention group (0.017) compared to the control group (0.047) (p-value unknown)  **ES -0.19** |  |  |  |

**S2 Table. Intervention effects on BMI/BMI z-score, physical activity behavior, sedentary behavior and nutrition behavior (continued).**

| **Authors** | **BMI/BMI z-score results** | **Physical activity behavior results** | **Sedentary behavior results** | **Nutrition behavior results** |
| --- | --- | --- | --- | --- |
| Centis et al. (2012) [34] | A decrease of in SDS BMI for the intervention group (-0.06) and an increase of for the control group (0.13) (p < 0.001)  **ES -0.19** | A larger increase in time spent in outdoor activities for the intervention group (3.70 h/week) compared to the control group (0.93 h/week) (p = 0.0155) **ES 0.49** | A decrease of in TV watching for the intervention group (0.96 h/week) and an increase for the control group (1.33 h/week) (p = 0.0044) **ES -0.38** | NA |
|  | A smaller increase in BMI for the intervention group (0.16 kg/m^2^) compared to the control group (0.75 kg/m^2^) (p < 0.001) **ES -0.27** | A smaller decrease in time spent in extra-school sports courses for the intervention group (-0.12 h/week) compared to the control group (-0.35 h/week) *(NR)* **ES 0.15** |  |  |
| Chomitz et al. (2010) [45] | A decrease in BMI z-score for the intervention group (-0.04) (no control group) (p < 0.001) **ES -0.04** | NA | NA | NA |
| Cong et al. (2012) [35] / Feng et al. (2016) [36] | A larger decrease in BMI percentile for age and gender for the intervention group  (-3.07) compared to the control group (-0.1) (ns) **ES -0.10** | NA | A smaller increase in screen time behavior for the intervention group (0.61 h/day) compared to the control group (β = -0.160) *(NR)* (p < 0.05) | A smaller increase in sugar-sweetened beverages for the intervention group (from 1.91 oz/day) compared to the control group (8.71 oz/day) (p < 0.05) **ES -0.42** |
| Crespo et al. (2012) [26] | An increase in BMI z-score for the intervention group (0.09) and a decrease for the control group (-0.03) (p = 0.83) **ES 0.11** | An increase in PA compared to other children of same age and sex for the intervention group (0.3) and the control group (0.28) (p = 0.74) **ES 0.02** | A larger decrease in frequency of TV viewing while getting ready for school for the intervention group (-0.52) compared to the control group (-0.01) (p = 0.92) **ES -0.41** | A smaller increase in fruit and vegetable intake for the intervention group (0.32 servings/day) compared to the control group (0.47 servings/day) (p = 0.55) **ES -0.05** |
|  | An increase in BMI percentile for the intervention group (2.47) and a decrease for the control group (-1.33) (p = 0.88) **ES 0.04** | A larger increase in number of sports participated in the past year for the intervention group (1.11 sports/year) compared to the control group (1.0 sports/year) (p = 0.035) **ES 0.10** |  | A smaller decrease in snack intake for the intervention group (-0.44 servings/day) compared to control group (-0.47 servings/day) (p = 0.38) **ES 0.01** |
|  |  |  |  | A smaller decrease in sugar-sweetened beverages consumption for the intervention group (-0.4 servings/day) compared to control group (-0.49 servings/day) (p = 0.34)  **ES 0.06** |
|  |  |  |  | A smaller increase in water consumption for the intervention group (0.22 servings/day) compared to control group (0.43 servings/day) (p = 0.41) **ES 0.01** |
| Engelen et al. (2013) [27] | NA | *Break time PA:*  No difference in LPA during break time for the intervention group and a decrease for the control group (-0.1 min/break time) (p = 0.79)  **ES 0.00** | Break time SB:  A decrease in sedentary behavior for the intervention group (-1.1 min/break time) compared to an increase for the control group (1.0 min/break time) (p = 0.01)  **ES -0.02** | NA |

**S2 Table. Intervention effects on BMI/BMI z-score, physical activity behavior, sedentary behavior and nutrition behavior (continued).**

| **Authors** | **BMI/BMI z-score results** | **Physical activity behavior results** | **Sedentary behavior results** | **Nutrition behavior results** |
| --- | --- | --- | --- | --- |
| Engelen et al. (2013) [27] |  | An increase in MVPA during break time for the intervention group (0.4 min/ break time) compared to a decrease for the control group (-1.4 min/break time) (p = 0.006)  **ES 0.19** |  |  |
|  |  | *Total PA:*  A decrease daily LPA for the intervention group (-2.1 min/day) and an increase for the control group (2.7 min/day) (p = 0.14)  **ES -0.15** | *Total SB:*  A smaller decrease in minutes per day of sedentary behavior for the intervention group (-3.2 min/day) compared to the control group (-3.5 min/day) (p = 0.99)  **ES 0.0** |  |
|  |  | An increase daily MVPA for the intervention group (0.5 min/day) and a decrease for the control group (-1.0 min/day) (p = 0.19)  **ES 0.0** |  |  |
| Hopper et al. (1996) [37] | NA | NA | NA | A smaller increase in cholesterol intake for the intervention group (35.06 mg/weekday) compared to the control group (51.78 mg/weekday) (ns) |
|  |  |  |  | A smaller increase in saturated fat intake for the intervention group (1.98 mg/weekday) compared to the control group (2.61 mg/weekday) (ns) |
|  |  |  |  | An increase of in vegetable and fruit servings per day for the intervention group (0.35 servings/day) and a decrease for the control group (-0.25 servings/day) (p < 0.05)  **ES 0.35** |
|  |  |  |  | A smaller decrease in grain and cereal servings per day for the intervention group (-0.48 servings/day) compared to the control group (-0.83) (ns) |
| Jiang et al. (2007) [28] | A smaller increase in BMI for the intervention group (0.6 kg/m2) compared to the control group (2.8 kg/m2) (p < 0.01)  **ES -0.79** | NA | NA | NA |
| Kain et al. (2004) [38] | *Boys:*  No change in BMI of boys in the intervention group compared to an increase in BMI of the boys in the control group (0.3 kg/m^2^) (p < 0.001) **ES -0.08** | NA | NA | NA |

**S2 Table. Intervention effects on BMI/BMI z-score, physical activity behavior, sedentary behavior and nutrition behavior (continued).**

| **Authors** | **BMI/BMI z-score results** | **Physical activity behavior results** | **Sedentary behavior results** | **Nutrition behavior results** |
| --- | --- | --- | --- | --- |
| Kain et al. (2004) [38] | A larger decrease in BMI z-score of boys in the intervention group (-0.12) compared to the decrease in BMI z-score of boys in the control group (-0.02) (P < 0.001) **ES -0.10** |  |  |  |
|  | *Girls:*  A larger increase in BMI of girls in the intervention group (0.03 kg/m^2^) compared to the BMI of girls in the control group (0.02 kg/m^2^) (p = 0.60) **ES 0.03** |  |  |  |
|  | A smaller decrease in BMI z-score of girls in the intervention group (-0.04) compared to the BMI z-score of girls in the control group (-0.07) (p = 0.49) **ES 0.03** |  |  |  |
|  | *Total group:*  No change in BMI for the intervention group compared an increase for the control group (0.3 kg/m^2^) (sign) |  |  |  |
|  | A decrease in BMI z-score for the intervention group compared to no change for the control group (p < 0.001) |  |  |  |
| Li et al. (2014) [39] | A decrease in BMI for the intervention group (0.02 kg/m²) compared to an increase for the control group (0.41 kg/m²)  (p < 0.001) **ES -0.10** | An increase in total MVPA for the intervention group (8.9 min/day) compared to a decrease of for the control group (-13.8 min/day) (p < 0.001) **ES 0.41** | NA | NA |
| Manios et al. (1999) [29] | A smaller increase in BMI for the intervention group (0.7 kg/m²) compared to the control group (1.8 kg/m²) (p = 0.001)  **ES -0.48** | A larger increase in leisure-time MVPA for the intervention group (2.0 h/week) compared to the control group (0.4 h/week) (p < 0.0005) **ES 0.98** | NA | A smaller increase in energy intake for the intervention group (269.7 kcal/day) compared to the control group (296.8 kcal/day) (ns) **ES 0.00** |
|  |  |  |  | A smaller increase in total fat intake for the intervention group (8.3 g/day) compared to the control group (9.0 g/day) (ns) **ES -0.02** |
|  |  |  |  | A larger increase in cholesterol intake for the intervention group (71.5 mg/day) compared to the control group (22.5 mg/day) (ns) **ES 0.32** |
|  |  |  |  | A smaller increase in protein intake for the intervention group (6.4 g/day) compared to the control group (7.8 g/day) (ns) **ES -0.06** |

**S2 Table. Intervention effects on BMI/BMI z-score, physical activity behavior, sedentary behavior and nutrition behavior (continued).**

| **Authors** | **BMI/BMI z-score results** | **Physical activity behavior results** | **Sedentary behavior results** | **Nutrition behavior results** |
| --- | --- | --- | --- | --- |
| Manios et al. (1999) [29] |  |  |  | A larger increase in carbohydrate intake for the intervention group (38.4 g/day) compared to the control group (36.3 g/day) (ns) **ES 0.02** |
|  |  |  |  | A smaller increase in fiber intake for the intervention group (0.05 g/day) compared to the control group (0.5 g/day) (ns) **ES -0.27** |
| Müller et al. (2001) [46] | NA | An increase in percentage children reporting daily physical activities for the intervention group (18% more children) (no control group) (p < 0.05) | A decrease in hours per day of TV watching for the intervention group (-0.3 h/days) (no control group) (p < 0.05) | An increase by 50% in the consumption of fruit and vegetables daily for the intervention group (20% more children) (no control group) (p < 0.05) |
| Prelip et al. (2012) [40] | NA | NA | NA | A smaller decrease in times fruit eaten per day for the intervention group (-0.04 times/day) compared to the control group (--0.29 times/day) (ns) **ES 0.18** |
|  |  |  |  | An increase in vegetables eaten per day for the intervention group (0.13 times/day) and a decrease for the control group (from --0.20 times/day) (ns) **ES 0.20** |
| Sanigorski et al. (2008) [41] | A larger increase in BMI for the intervention group (1.7 kg/m^2^) compared to the control group (1.3 kg/m^2^) (p = 0.20) **ES 0.14** | NA | NA | NA |
|  | A larger decrease in BMI z-score for the intervention group (-0.09) compared to the control group (-0.02) (p = 0.04) **ES -0.08** |  |  |  |
| Sharma et al. (2016) [42] | NA | NA | NA | An increase in fruit intake for the intervention group (0.13 cups/1000 kcal per day) and a decrease for the control group  (-0.01 cups/1000 kcal per day) (p = 0.046)  **ES 0.17** |
|  |  |  |  | A larger increase in vegetable intake for the intervention group (0.09 cups/1000 kcal per day) compared to the control group (0.03 cups/1000 kcal per day) (p = 0.049) **ES 0.21** |
|  |  |  |  | A larger decrease in added sugar intake for the intervention group (-063 tsp/1000 kcal per day) compared to the control group  (-0.06 tsp/1000 kcal per day) (p = 0.014)  **ES -0.21** |

**S2 Table. Intervention effects on BMI/BMI z-score, physical activity behavior, sedentary behavior and nutrition behavior (continued).**

| **Authors** | **BMI/BMI z-score results** | **Physical activity behavior results** | **Sedentary behavior results** | **Nutrition behavior results** |
| --- | --- | --- | --- | --- |
| Sharma et al. (2016) [42] |  |  |  | A larger decrease in estimated percent of daily kilocalories from sugar beverages for the intervention group (-0.88%) compared to the control group (-0.33%) (p = 0.106)  **ES -0.16** |
|  |  |  |  | A larger increase in total fiber intake for the intervention group (0.82 grams/1000 kcal/day) compared to the control group (0.29 grams/1000 kcal/day) (p = 0.051)  **ES 0.17** |
|  |  |  |  | A larger decrease in total fat intake for the intervention group (-1.22 grams/1000 kcal/day) compared to the control group  (-0.41 grams/1000 kcal/day) (p = 0.188)  **ES -0.13** |
|  |  |  |  | A decrease in daily kilocalories for the intervention group (-17.36 kcal/day) and an increase for the control group (45.14 kcal/day) (p = 0.228) **ES -0.11** |
|  |  |  |  | A larger increase in whole grains intake for the intervention group (0.07 ounce/1000 kcal/day) compared to the control group (-0.02 ounce/1000 kcal/day) (p = 0.129)  **ES 0.15** |
| Siegrist et al. (2013) [30] | A larger increase in BMI for the intervention group (0.7 kg/m^2^) compared to the control group (0.6 kg/m^2^) (p = 0.165) **ES 0.03** | A larger increase in the amount of days per week being active at least 60 min/day for the intervention group (0.5 days/week) compared to the control group (0.1 days/week) (p = 0.109) **ES 0.20** | NA | NA |
|  | A larger increase in SDS-BMI for the intervention group (0.06) compared to the control group (0.01) (p = 0.057) **ES 0.05** |  |  |  |
| Treu et al. (2015) [43] | A larger increase in BMI for the intervention group (0.6 kg/m^2^) compared a decrease for the control group (-0.3 kg/m^2^) (p = 0.330)  **ES 0.07** | NA | NA | NA |
|  | A larger increase in BMI z-score for the intervention group (0.06) compared to the control group (0.01) (p = 0.157) **ES 0.05** |  |  |  |

**S2 Table. Intervention effects on BMI/BMI z-score, physical activity behavior, sedentary behavior and nutrition behavior (continued).**

| **Authors** | **BMI/BMI z-score results** | **Physical activity behavior results** | **Sedentary behavior results** | **Nutrition behavior results** |
| --- | --- | --- | --- | --- |
| Waters et al. (2018) [32] | A smaller BMI for the intervention group (17.82 kg/m^2^) compared to the control group (17.95 kg/m^2^) at follow-up (mean difference: -0.16 kg/m^2^) (p = 0.36) | A higher percentage of children in the intervention group performing active games at lunchtime (73.1%) compared to the control group (69.7%) at follow-up (OR: 1.51, 95% CI: 0.84-2.69) | A higher percentage of children in the intervention group watching TV 2 hours or less per week day (82.1%) compared to the control group (79.8%) at follow-up (OR: 0.97, 95% CI: 0.75-1.25) | A higher number of fruit serves for the intervention group (2.48) compared to the control group (2.37) at follow-up (mean difference: 0.19) (p = 0.05) |
|  |  |  |  | A higher number of vegetable serves for the intervention group (2.15) compared to the control group (2.03) at follow-up (mean difference: 0.13) (p = 0.10) |
|  |  | A higher percentage of children in the intervention group being outside 2 hours or more yesterday (11.7%) compared to the control group (11.2%) at follow-up (OR: 1.33, 95% CI: 0.75-2.37) | A higher percentage of children in the intervention group watching TV 2 hours or less per weekend day (61.0%) compared to the control group (60.3%) at follow-up (OR: 1.06, 95% CI: 0.89-1.27) | A higher percentage of children in the intervention group consuming soft drink per day (28.0%) compared to the control group (34.0%) at follow-up (OR: 0.89, 95% CI: 0.60-1.32) |
|  | A smaller BMI z-score for the intervention group (0.68) compared to the control group (0.72) at follow-up (mean difference: -0.05) (p = 0.44) | A lower percentage of children in the intervention group being outside 2 hours or more on a weekend day (54.4%) compared to the control group (56.2%) at follow-up (OR: 0.88, 95% CI: 0.63-1.24) |  | A higher percentage of children in the intervention group consuming any fruit juice per day (61.4%) compared to the control group (67.6%) at follow-up (OR: 0.86, 95% CI: 0.66-1.13) |
|  |  |  |  | A higher percentage of children in the intervention group consuming 2 or more glasses of water per day (94.3%) compared to the control group (90.7%) at follow-up (OR: 1.33, 95% CI: 0.78-2.30) |
| Xu et al. (2015) [31] | A larger decrease in BMI for the intervention group (-0.32 kg/m²) compared to the control group (-0.29 kg/m²) (p = 0.09)  **ES -0.01** | A higher percentage of children in the intervention group improved in frequency jogging/running (46.0%) compared to the control group (32.4%) (OR: 1.55, 95% CI: 1.18-2.02) | A higher percentage of children in the intervention group reduced the time viewing TV or using computers (49.4%) compared to the control group (41.0%) (OR: 1.41, 95%CI: 1.09-1.84) | A higher percentage of children in the intervention group decreased their red meat consumption (46.1%) compared to the control group (35.0%) (OR: 1.50, 95% CI: 1.15-1.95) |
|  |  | A higher percentage of children of the intervention group improved in frequency walking (46.9%) compared to the control group (45.7%) (OR: 0.98, 95% CI: 0.74-1.25) |  | A higher percentage of children in the intervention group decreased their fried snack consumption (29.1%) compared to the control group (27.4%) (OR: 1.08, 95% CI: 0.81-1.44) |
|  |  | A higher percentage of children of the intervention group improved in frequency ball playing (40.0%) compared to the control group (35.8%) (OR: 1.21, 95% CI: 0.93-1.58) |  | A lower percentage of children in the intervention group decreased their soft drink consumption (26.4%) compared to the control group (28.2%) (OR 0.89; 95% CI: 0.67-1.19) |
|  |  | A higher percentage of children of the intervention group improved in their commuting mode to school (28.9%) compared to the control group (16.5%) (OR: 2.24, 95% CI: 1.47-3.40) |  | A higher percentage of children in the intervention group increased their vegetable consumption (48.6%) compared to the control group (47.1%) (OR: 1.20, 95% CI: 0.92-1.55) |

**S2 Table. Intervention effects on BMI/BMI z-score, physical activity behavior, sedentary behavior and nutrition behavior (continued).**

| **Authors** | **BMI/BMI z-score results** | **Physical activity behavior results** | **Sedentary behavior results** | **Nutrition behavior results** |
| --- | --- | --- | --- | --- |
| Xu et al. (2017) [47] | A smaller increase in BMI for the intervention group (0.06 kg/m^2^) compared to the control group (0.08 kg/m^2^) (p < 0.001)  **ES -0.06** | NA | NA | NA |
|  | A decrease in BMI z-score for the intervention group (-0.11) compared to an increase for the control group (0.03) (p < 0.001)  **ES -0.10** |  |  |  |

BMI = Body Mass Index, NA = Non-Applicable, ns = non-significant (p ≥ 0.05), sign = significant, NR = Not Reported, ES = Effect Size (Cohen’s d), PA = Physical Activity Behavior, MVPA = moderate-to-vigorous physical activity behavior, LPA = light physical activity behavior. OR = Odds Ratio, CI = Confidence Interval.
